# Development and some applications of enzyme-linked immunosorbent assay system for murine macrophage inflammatory protein-2 (MIP-2)

**DOI:** 10.1155/S0962935196000294

**Published:** 1996-06

**Authors:** H. Ochiai, S. Sakai, T. Kogure, T. Hirabaytashi, K. Nakajima, K. Terasawa

**Affiliations:** 1 Department of Human Science Faculty of Medicine Toyama Medical and Pharmaceutical University 2630 Sugitani Toyama 930-01 Japan; 2 Department of japanese Oriental Medicine Faculty of Medicine Toyama Medical and Pharmaceutical University 2630 Sugitani Toyama 930-01 Japan; 3 Department of Virology Medical School Nagoya City University Mizuho-machi Nagoya 467 Japan

## Abstract

We attempted to establish an enzyme-linked immunosorbent assay (ELISA) system by preparation of recombinant murine MIP-2 and its rabbit antibodies. A fusion construct of MIP-2 to protein A was used to enable easy purification as well as the generation of a sufficiently large antibody response. The specificity of antibody was confirmed by Western blotting analysis of 20-h conditioned medium from lipopolysaccharide (LPS)-stimulated RAW264.7 cells, a murine macrophage cell line; antibody gave a single band with a molecular weight of approximately 6000, which is identical to that of murine MIP-2 reported previously. Biotin–streptavidin sandwich ELISA could detect quantitatively MIP-2 at concentration range of 20 to 1000 pg/ml. In some applications of this ELISA system, time-related production of MIP-2 and inhibitory effect of dexamethasone on its production have been demonstrated in LPS-stimulated RAW264.7 cells. Thus, ELISA system established in this study is considered to be a useful tool to study MIP-2 response in various inflammation models in mice.


Mediators of Inflammation, 5, 206–209 (1996)

### Development and some applications of enzyme-linked immunosorbent assay system for murine macrophage inflammatory protein-2 (MIP-2)

H. Ochiai,[1,CA] S. Sakai,[2] T. Kogure,[2]
T. Hirabaytashi,[2] K. Nakajima[3] and K. Terasawa[2]

Departments of [1]Human Science, and [2]Japanese Oriental Medicine, Faculty of Medicine, Toyama Medical and Pharmaceutical University, 2630 Sugitani, Toyama 930-01, Japan, and [3]Department of Virology, Medical School, Nagoya City University, Mizuho-machi, Nagoya 467, Japan

[CA]Corresponding Author

**We attempted to establish an enzyme-linked immunosorbent assay (ELISA) system by preparation of recombinant murine MIP-2 and its rabbit antibodies. A fusion construct of MIP-2 to protein A was used to enable easy purification as well as the generation of a sufficiently large antibody response. The specificity of antibody was confirmed by Western blotting analysis of 20-h conditioned medium from lipopolysaccharide (LPS)-stimulated RAW264.7 cells, a murine macrophage cell line; antibody gave a single band with a molecular weight of approximately 6 000, which is identical to that of murine MIP-2 reported previously. Biotin–streptavidin sandwich ELISA could detect quantitatively MIP-2 at concentration range of 20 to 1 000 pg/ml. In some applications of this ELISA system, time-related production of MIP-2 and inhibitory effect of dexamethasone on its production have been demonstrated in LPS-stimulated RAW264.7 cells. Thus, ELISA system established in this study is considered to be a useful tool to study MIP-2 response in various inflammation models in mice.**

**Key words:** Antibody-sandwich ELISA, Lipopolysaccharide, Murine macrophage inflammatory protein-2, RAW264.7 cells

### Introduction

Neutrophil accumulation is an important characteristic of inflammation and modulates various inflammatory reactions.[1] Recently, novel neutrophil chemotactic cytokines (chemokines), have been found in the conditioned medium (CM) of various cells including monocytes/macrophages, fibroblasts, endothelial cells and epithelial cells in response to the stimulation with lipopolysaccharide (LPS) as well as several inflammatory cytokines such as IL-1 and tumour necrosis factor.[2–7] Several studies on the pathogenesis of inflammatory diseases have suggested that chemokine production is the main cause of the local accumulation of neutrophils and also their activation.[8,9] cDNA of murine macrophage inflammatory protein 2 (MIP-2), a potent chemotactic agent, has been cloned from murine macrophage RAW 264.7 cells[10,11] and MIP-2 is considered to be a murine counterpart of chemokine superfamily based on the closest homology of cDNA.[2] Although Greenberger *et al.*[13] have touched upon a murine model of bacteria infection, MIP-2 response to various inflammatory stimuli has not been studied in detail because of the difficulty of development of the assay system. Thus, we attempted to establish an enzyme-linked immunosorbent assay (ELISA) system for murine MIP-2, and based on the development of the assay system, we applied this ELISA to examine the time-related MIP-2 production and inhibitory effect of dexamethasone (DX) in LPS-stimulated RAW264.7 cells.

### Materials and Methods

RAW 264.7 cells, used throughout this study, were obtained from American Type Culture Collection and cultured in Dulbecco's modified Eagle's minimal essential medium (DMEM). MIP-2 cDNA was amplified under reverse transcriptase–polymerase chain reaction in the reaction mixture containing purified mRNA from RAW264.7 cells, which were cultured in the presence of 1 μg/ml of LPS of *Escherichia coli* (O127:B8, Difco, Detroit, MI) for 20 h at 37°C, and primers matching to amplify the whole length of MIP-2 mRNA (221 bases from alanine- to asparagine-encoding regions).[10,11] Murine MIP-2 was expressed as a fusion protein with staphylococcal protein A by inserting cDNA of MIP-2 into *Hin*dIII and *Sma*I cutting sites of plasmid vector pRIT12.[12] The construct was confirmed by sequencing. All recombinant DNA techniques were performed essentially as described previously.[12] One of the reasons for the construction of protein A–MIP-2 expression vector is to enable easy purification of recombinant MIP-2 by IgG column. Another reason is that the molecular

© 1996 Rapid Science Publishers weight (MW) of MIP-2 itself (approximately 6000)[10,11] is too small to induce the sufficient antibody production. Indeed, antibody against CINC, a rat counterpart of chemokine superfamily, has been prepared by immunization with keyhole limpets haemocyanine-conjugated CINC in the previous study.[9]

### Results and Discussion

When the lysates of *E. coli* carrying expression vector were applied to affinity chromatography on IgG Sepharose GFF column (Pharmacia, Uppsala, Sweden), the binding fraction on the column showed a single band with MW of approximately 50 000 in sodium dodecyl sulphate polyacrylamide gel electrophoresis (SDS-PAGE)[14] (data not shown). This finding indicates that a single application to the column is sufficient for the purification of recombinant MIP-2 fused with protein A of which MW is 42 000.[15] Thus, the recombinant MIP-2 was injected intracutaneously into the rabbit for the preparation of hyperimmune anti-MIP-2 serum (initially injected with complete Freund's adjuvant followed by boosts with incomplete adjuvant every 2 weeks), and then anti-MIP-2 IgG was purified using protein G column (Pharmacia, Uppsala, Sweden). Some portions of purified anti-MIP-2 IgG were conjugated with CNBr-activated Sepharose 6MB (Pharmacia, Uppsala, Sweden) to examine its specificity as follows. The lyophilized 20-h CM from LPS-stimulated RAW264.7 cells was applied to the anti-MIP-2 IgG-conjugated Sepharose column, and the binding fraction on the column was analysed by SDS-PAGE. Alternatively, Western blotting analyses[16] of the CM were carried out. As shown in Fig. 1, anti-MIP-2 IgG reacted with single molecule with MW of 6 000, which is identical to that of murine MIP-2,[10,11] in the CM from LPS-stimulated cells, but not reacted with the CM from unstimulated cells, confirming the specificity of anti-MIP-2 IgG.

Based on these findings, murine MIP-2 detection system was prepared as an antibody sandwich ELISA in which unlabelled (1.5 μg/ml) and biotinylated (5 μg/ml) rabbit anti-murine MIP-2 IgG were used as capture and second-layer antibodies, respectively, followed by addition of peroxidase-coupled streptavidin and substrate for colour development as described previously.[17] For standardization of MIP-2 concentrations, MIP-2 was purified by affinity absorbent as above and used after determination of protein concentrations by Lowry's method.[18] As shown in Fig. 2, a lineal relation was obtained between concentration of the purified MIP-2 and absorbance at 490 nm in the concentration range of 20 to 1 000 pg/

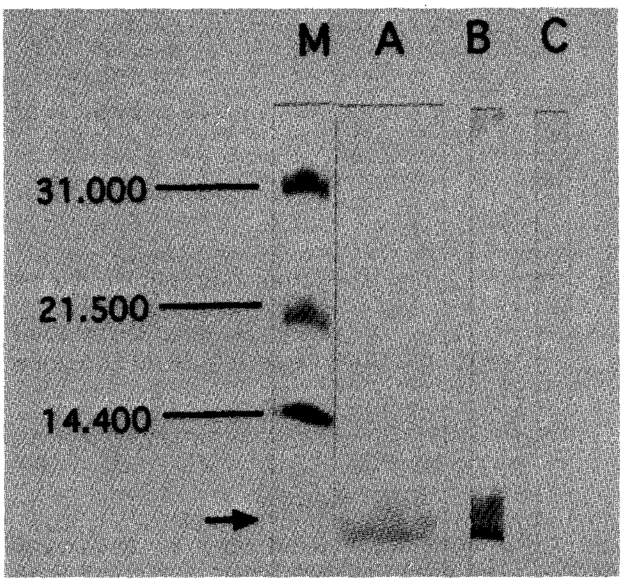

FIG. 1. Western blotting analysis of purified MIP-2. The 20-h CM from LPS-stimulated RAW264.7 cells was applied to anti-MIP-2 IgG-conjugated Sepharose column. The lyophilize binding fraction was analysed by SDS-PAGE on 15–25% gradient gel (Daiichi Pure Chemicals Co. Ltd, Tokyo, Japan) followed by visualization of protein with dye (lane A). Alternatively, the lyophilized CM from LPS-stimulated (lane B) or -unstimulated (lane C) cells was applied directly to SDS-PAGE as above and then transferred electrophoretically to a nitrocellulose filter. SDS-PAGE was carried out in the presence of 2-mercaptoethanol. Thereafter, the immunochemical detection of antigenic protein bands on filter was carried out using biotinylated anti-MIP-2 IgG and horseradish peroxidase-conjugated streptavidin followed by the addition of substrate to visualize protein bands. Electrophoresis calibration kit (Pharmacia, Uppsala, Sweden) was used as MW standards (31 000, 21 500 and 14 400) (lane M).

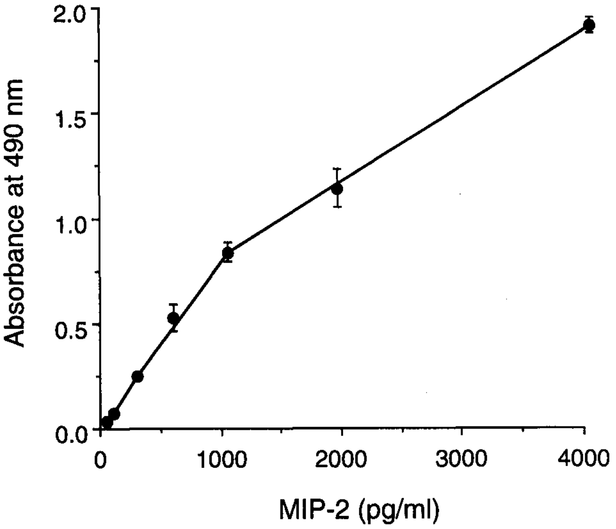

FIG. 2. Standardization for concentrations of murine MIP-2 in ELISA. Purified MIP-2 (see Fig. 1 legend) was diluted with phosphate buffered saline and applied to ELISA in which anti-MIP-2 IgG (0.15 mg/ml) and biotinylated anti-MIP-2 IgG (0.05 mg/ml) were used for capture and second layer antibodies, respectively, followed by addition of peroxidase-coupled streptavidin and substrate for colour development. The reaction was stopped with 6 N $H_2SO_4$ and the optical density at 490 nm was recorded. Triplicate wells were used for each experimental point to calculate the mean (closed circle) ± S.E. (thin bar).

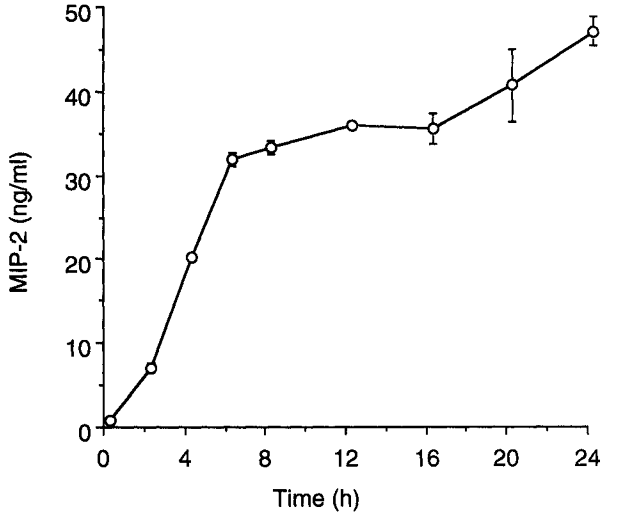

FIG. 3. Time-related production of MIP-2 in LPS-stimulated RAW264.7 cells. The cells were inoculated into a 96-well microplate at a density of 1 × $10^5$ cells/well. After culture overnight at 37°C, the cells were washed once with serum-free DMEM and further cultured in the presence of LPS (1 μg/ml). This time was designated as 0 h. At the indicated times (abscissa), the CM was collected to detect MIP-2 by ELISA. Triplicate wells were used for each experimental point to calculate the mean (closed circle) ± S.E. (thin bar).

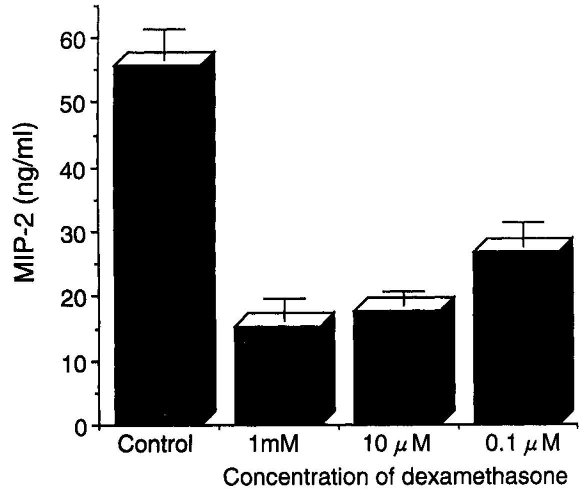

FIG. 4. Inhibitory effect of dexamethasone on the production of MIP-2 in LPS-stimulated RAW264.7 cells. The cells were cultured in the presence of both LPS and dexamethasone at a dose range of 0 to $10^{-7}$ M (abscissa). Thereafter, 20-h CM was collected to measure MIP-2 concentrations. Triplicate wells were used for each experimental point to calculate the mean (thick bar) ± S.E. (thin bar). Cell preparation and culture condition were described in legend for Fig. 3.

ml, indicating that this ELISA system could detect quantitatively MIP-2.

When this ELISA system was applied to detect the time-related production of MIP-2 in LPS-stimulated RAW264.7 cells, MIP-2 was undetectable (less than 20 pg/ml) at 0 h. At 2 h, a low but recognizable level of MIP-2 (6 ng/ml) was detectable, and then this level increased sharply to 32 ng/ml from 4 to 8 h and then gradually increased to 20 h at which the plateau level attained (Fig. 3). In the case of LPS-stimulated P388D1 cells, which are other murine macrophage-like cells, MIP-2 was also detected in 20-h CM at a comparable level to that in RAW264.7 cells (data not shown).

Furthermore, because DX is a well-known inhibitor on certain chemotactic factors such as human interleukin-8,[19] it was examined whether this is the case in MIP-2 response. As shown in Fig. 4, DX inhibited dose-dependently MIP-2 production in a dose range of $10^{-3}$ to $10^{-7}$ M. These data indicate that MIP-2 production might be regulated DX-sensitive factors in accordance with the previous report.[19]

In summary, recombinant murine MIP-2 was expressed as a fusion protein with staphylococcal protein A to enable easy purification of recombinant MIP-2 and enhance antigenic potential without the help of a carrier protein. This recombinant MIP-2 was considered to be sufficient for the preparation of antibody from the aspects of specificity and antigenicity, leading to establishment of biotin–streptavidin sandwich ELISA system for murine MIP-2. In some applications of this ELISA system, time-related MIP-2 production and inhibitory effect of DX on its production could be detected in a murine macrophage cell line in response to LPS stimulation. Thus, it is suggested that this assay system should be useful for the studies of MIP-2 response in various inflammatory models in mice.

### References

1. Gallin JI, Wright DG. Role of secretory events in modulating human neutrophil chemotaxis. *J Clin Invest* 1987; **62**: 1364–1374.
2. Driscoll KE. Macrophage inflammatory proteins: biology and role in pulmonary inflammation. *Exp Lung Res* 1994; **20**: 473–490.
3. Gregory H, Young J, Schroder J-M, Morowietz U, Christophers E. Structure determination of a human lymphocyte derived neutrophil activating peptide (LYNAP). *Biochem Biophys Res Commun* 1988; **151**: 883–890.
4. Larsen CG, Anderson AO, Appella E, Oppenheim JJ, Matsushima K. The neutrophil-activating protein (NAP-1) is also chemotactic for T-lymphocytes. *Science* 1989; **243**: 1464–1466.
5. Waltz A, Peveri P, Aschauer H, Baggiolini M. Purification and amino acid sequencing of NAF, a novel neutrophil-activation factor produced by monocytes. *Biochem Biophys Res Commun* 1987; **149**: 755–761.
6. Yoshimura T, Matsushima K, Tanaka S, Robinson EA, Appella E, Oppenheim JJ, Leonard EJ. Purification of a human monocyte-derived neutrophil chemotactic factor that has peptide sequence similarity to other host defense cytokines. *Proc Natl Acad Sci USA* 1987; **84**: 9233–9237.
7. Watanabe K, Konishi K, Fujioka M, Kinoshita S, Nakagawa H. The neutrophil chemoattractant produced by the rat kidney epitheloid cell line NRK-52E is a protein related to the KC/gro protein. *J Biol Chem* 1989; **264**: 19559–19563.
8. Watanabe K, Koizumi F, Kurashige Y, Tsurufuji S, Nakagawa H. Rat CINC, a member of the interleukin-8 family, is a neutrophil-specific chemoattractant *in vivo*. *Exp Mol Pathol* 1991; **55**: 30–37.
9. Iida M, Watanabe K, Tsurufuji M, Takahashi K, Iizuka Y, Tsufuji S. Level of neutrophil chemotactic factor CINC/gro, a member of the interleukin-8 family, associated with lipopolysaccharide-induced inflammation in rats. *Infect Immun* 1992; **60**: 1268–1272.
10. Wolpe SD, Sherry B, Jueres D, Davatelis G, Yurt RW, Cerami A. Identification and characterization of macrophage inflammatory protein 2. *Proc Natl Acad Sci USA* 1989; **86**: 612–616.
11. Tekamp-Olson P, Gallegos C, Bauer D, McClain J, Sherry B, Fabre M, Van Deventer S, Cerami A. Cloning and characterization of cDNA for murine macrophage inflammatory protein 2 and its human homologues. *J Exp Med* 1990; **172**: 911–919.
12. Shibata F, Kato H, Konishi K, Okumura A, Ochiai H, Nakajima K, Al-Mokdad M, Nakagawa H. Differential changes in the concentrations of cytokine-induced neutrophil chemoattractant (CINC)-1 and CINC-2 in exudate during rat lipopolysaccharide-induced inflammation. *Cytokine* 1996; **8**: 222–226.
13. Greenberger MJ, Strieter RM, Kunkel SL, Danforth JM, Goodman RE, Standiford TJ. Neutralization of IL-10 increases survival in a murine model of *Klebsiella* pneumonia. *J Immunol* 1995; **155**: 722–729.
14. Laemmli UK. Cleavage of structural proteins during the assembly of the head of bacteriophage T4. *Nature* 1970; **227**: 680–685.
15. Björk I, Petersson BÅ, Sjöquist J. Some physicochemical properties of protein A from *Staphylococcus aureus*. *Eur J Biochem* 1972; **29**: 579–584.
16. Towbin H, Staehelin T, Gordon J. Electrophoretic transfer of proteins from polyacrylamide gels to nitrocellulose sheets: procedure and some applications. *Proc Natl Acad Sci USA* 1979; **76**: 4350–4354.
17. Nakagawa H, Ikesue A, Katoh H, Debuchi H, Watanabe K, Tsurufuji S, Naganawa M, Mitamura M. Changes in the levels of rat interleukin-8/CINC and gelatinase in the exudate of carrageenin-induced inflammation in rats. *J Pharmacobio-Dyn* 1992; **15**: 461–466.
18. Lowry OH, Rosenbrough NJ, Farr AL, Randall RJ. Protein measurement with the folin phenol reagent. *J Biol Chem* 1951; **193**: 265–275.
19. Mukaida N, Gussella GL, Kasahara T, Ko Y, Zacchariae COC, Kawai T, Matsushima K. Molecular analysis of the inhibition of interleukin-8 by dexamethasone in a human fibrosarcoma cell line. *Immunology* 1992; **75**: 674–679.

ACKNOWLEDGEMENT. This work was supported in part by the grant for the research of Oriental Medicine from Tokyo Metropolitan Government.

**Received 30 January 1996;**
**accepted in revised form 11 March 1996**
